# Potential Impact of Updated Bayesian Deduction in Medicine: Application to Colonoscopy Prioritization

**DOI:** 10.3390/cancers17233845

**Published:** 2025-11-29

**Authors:** Pierre Collet, Felipe Quezada-Diaz, Carla Taramasco

**Affiliations:** 1 ITISB, Facultad de Ingeniería, Universidad Andres Bello, Viña del Mar, Chile 2520000, Chile; carla.taramasco@unab.cl; 2 Unidad de Coloproctología, Servicio de Cirugía, Complejo Asistencial Doctor Sotero del Rio, Santiago 8550000, Chile; ffquezad@gmail.com; 3 Centro para la Prevención y Control del Cáncer (CECAN), Viña del Mar 2520000, Chile

**Keywords:** updated bayesian deduction, colorectal cancer screening, faecal immunochemical test, Resource Allocation, Evidence-Based Medicine

## Abstract

**Background/Objectives:** This study presents and explores the potential of Updated Bayesian Deduction (UBD) using colorectal cancer (CRC) detection and prioritisation as a case example. Colorectal cancer (CRC) is a leading cause of cancer-related mortality worldwide, and its prognosis strongly depends on early detection and timely treatment. In Chile, colonoscopy waiting lists for *symptomatic* patients in public hospitals can exceed one year, limiting access to early diagnosis and reducing survival rates. Traditional single-test screening strategies, such as a single faecal immunochemical test (FIT), often yield uncertain results, contributing to inefficiencies in resource allocation. **Methods:** We propose a deductive approach that integrates evidence from multiple sequential and independent FITs to dynamically update the posterior probability of CRC. A case study is analysed with this Updated Bayesian Deduction over a four-round FIT protocol to assess how this could improve risk stratification compared to standard symptoms-based screening. **Results:** Our mathematical model shows that over 85% of colonoscopies for symptomatic patients were not urgent. We then demonstrate that, if 4-FIT UBD were used to screen Chile’s Metropolitan Region population, only 96 out of 100,000 people would require an urgent colonoscopy to detect the 19.6 out of 100,000 individuals with CRC in this region. Many countries cannot afford a colonoscopy-based population screening, such as what is performed in Germany. Performing 4x FITs + a very small number of colonoscopies would be much more affordable and would get more countries to adopt general CRC screening. **Conclusions:** In countries with limited colonoscopy availability, such as Chile, where symptomatic patients can wait over a year for treatment in public hospitals, implementing a UBD-based strategy could drastically reduce costs and optimise the use of resources. This would improve access to colonoscopies for critical cases and ultimately enhance five-year survival rates. These findings highlight UBD as a promising approach for evidence-based precision medicine in CRC screening and prioritisation that is both explainable and adaptable.

## Simple Summary

Traditional single-test strategies using faecal immunochemical tests (FIT) for the detection of colorectal cancer can often yield uncertain results, leading to unnecessary colonoscopies or missed diagnoses. In this paper, we show how when applied to four consecutive FITs, Updated Bayesian Deduction (UBD) indicates that colonoscopies were unnecessary for over 85% of symptomatic patients and over 98% of asymptomatic patients. This approach also dynamically stratifies patient risk. UBD could also use other indicators to better assess cancer probability. This approach represents an important step towards explainable, evidence-based precision medicine. In Chile, for example, where waiting times for colonoscopies for symptomatic patients in public hospitals can exceed one year, this method could prioritise those with the highest likelihood of advanced neoplasia, thereby reducing delays for critical cases and potentially saving lives.

## 1. Introduction

Colorectal cancer (CRC) is the second and third most common cancer in women and men [1] and currently the second leading cause of cancer death worldwide [2]. Its particularity is that colonoscopy, the gold standard for its diagnosis, is also the treatment for stage 0 colon cancers that have not grown beyond the inner lining of the colon, as it is possible to remove polyps or cancerous areas by a local excision during the colonoscopy. This dual use of colonoscopy means that even if CRC were diagnosed by another method, a patient with CRC should undergo a colonoscopy to evaluate the extent of the cancer and/or remove the cancerous areas that can be removed during the procedure.

CRC prognosis is strongly linked to early detection, yet screening and diagnostic strategies remain constrained by resource limitations, particularly in low- and middle-income countries. In Chile, the challenge is critical: colonoscopy waiting times for *symptomatic* patients in public hospitals often exceed one year, significantly delaying diagnosis and reducing survival rates. Current screening is performed by doctors who refer symptomatic patients to hospitals where a colonoscopy is typically prescribed. Consequently, many patients undergo unnecessary colonoscopies, while others at high risk remain undiagnosed because no global screening (even based on a single FIT) is proposed.

Indeed, on top of being invasive and not harmless, colonoscopies are expensive and resource-intensive: the cost of a colonoscopy ranges from around USD 100 to over USD 4500, depending on the country and whether it is performed in the private or public sector [3,4]. However, several other CRC detection methods exist that look for the presence of blood in stool [5], such as faecal immunochemical tests (FITs).

FITs are:

- Very simple to perform: A stool sample can be collected at home in a jar and sent to a lab without any prior preparation or diet;
- Very cheap: The reported average cost is USD 3.04, with a range from USD 0.83 to USD 6.41 per test [6]), meaning that a FIT costs around 100x less than a colonoscopy;
- Non-invasive: They do not have associated risks and do not require sedation or recovery time;
- Immediately available: There is no waiting list for this very cheap test.
- However, they are less precise in their diagnosis:
- They have a higher false-positive rate (lower specificity) because they test for occult blood in stool, which is not specific to CRC (haemorrhoids can also cause rectal bleeding):
- They have a higher false-negative rate (lower sensitivity) due to the intermittent bleeding nature of cancerous polyps (cf. Section 2.1 below).

Despite their lower quality (lower specificity and sensitivity), FITs have been widely studied [7,8,9,10,11,12]. For the general screening of asymptomatic patients, study [9] found across forty-four studies involving 92,447 participants, including 3034 CRC cases, a sensitivity of 73% (i.e., 27% false negatives) for people with stage 1 CRC, with a specificity of around 94% (i.e., 6% false positives).

However, it is important to note that most papers focus on using FITs to diagnose colorectal cancer. This paper aims at the opposite: to determine which people *do not* have CRC in order to reduce waiting lists. But for this purpose, the sensitivity of the test is too low. Suppose we want to screen a cohort of 1,000,000 people, of which 1000 have CRC, with a single FIT (as currently performed in France [13]) with 27% false negatives. This means that 270 persons will receive a falsely negative FIT, meaning that they will not be contacted for a colonoscopy even though they have CRC.

Upon reading the literature on FITs, we realised that their low sensitivity stems not so much from the tests themselves, but rather from the fact that polyp bleeding is intermittent and could also be caused by manipulation errors. This means that repeating several FITs on the same person may yield different results.

In this paper, we introduce the technique of Updated Bayesian Deduction (UBD) applied to multiple sequential FIT tests. This is a Bayesian statistical framework that combines evidence from repeated, independent tests to update disease prevalence in subsequent subcohorts. In this study, we demonstrate—using clinical data—that applying UBD to a four-round FIT protocol shows that urgent colonoscopies are unnecessary for over 85% of symptomatic patients. Moreover, statistical modelling on Chile’s Metropolitan Region indicates that effective population-level screening could be achieved with as few as 0.01% of the colonoscopies currently needed, representing a paradigm shift for all countries (including Chile) that do not propose global population screening for CRC.

In Germany, CRC screening involves offering a colonoscopy to all men over 50 and women over 55 [14]. In France, a single FIT is recommended [13]. In Chile, there is no screening programme, yet CRC is the fastest-increasing cancer, with studies showing that its prevalence could increase by 60% by 2030 [15].

The primary objective of this study is twofold: First, to evaluate the clinical and operational impact of UBD FIT-based screening and prioritisation strategies in colorectal cancer (CRC) detection; and second, to highlight the potential of these strategies as an interpretable, evidence-based tool for precision medicine.

Section 2 presents and explains the method using a simple example. Section 3 presents the application of 4-FIT UBD to two cases:

- CRC screening in Chile’s Metropolitan Region, where the CRC prevalence is 19.6/100,000, and the accepted FIT sensitivity and specificity from [9];
- A published symptomatic cohort for prioritisation purposes, representative of a hospital’s needs, where the CRC prevalence is much higher.

We end with a discussion and a conclusion.

## 2. Presentation of the Method

### 2.1. Bibliographic Analysis

In this paper, we posit that the irregularity of observed occult blood in patients with confirmed CRC means that different FITs on the same patient could yield different results: a FIT could be positive one day, negative the next day, and positive again the day after, depending on the stage of the CRC or other factors.

We base this statement on the following papers:

- Ref. [16] showed a nonuniform distribution of occult blood in faeces;
- Ref. [17] studies bleeding patterns in colorectal cancer;
- Ref. [18] concludes (from a very small sample study) that the immunochemical faecal occult blood test “*is unsuitable for the diagnosis of rectal cancer, due to a low sensitivity of the diagnostic test combined with a slight haemorrhage or intraintestinal degeneration of haemoglobin, inappropriate site and amount of stool sampling, poor preservation of the specimen”.*
- More recently, ref. [19] shows, on a larger cohort, “*a low concordance between daily consecutive [FIT] tests results*”.

The intermittent bleeding of polyps in the early stages of development is why a single negative FIT is not conclusive enough to remove a patient from a waiting list. This has resulted in hundreds of papers studying the validity of FITs for diagnosing colorectal cancer from a frequentist point of view. It is this intermittent bleeding that led us to propose to use the Updated Bayesian Deduction approach for exploring the potential of performing several FITs within a short timeframe, cf. article [20] for a philosophical explanation of the difference between Bayesian inductive inference and Bayesian deductive inference.

The use of Bayesian Inference for inverting probabilities is certainly not a novel technique, as it has been described as early as 1763 by Bayes [21] and in 1771 by Laplace [22]. In this paper, we propose exploring the potential benefits of using Bayes’ theorem to create subcohorts with updated prevalence, with the aim of amplifying the value of data collected from previous clinical trials. The approach is not common and is not clearly found in books specialising in Bayesian statistics, such as references [23,24], probably because it is deductive and not inductive. Articles [25,26] show an analysis of its inductive inference but in this article, we remain within the realm of deductive inference. Article [27] is very interesting as it discusses how the independence of iterative tests can be measured with at least 4 tests. [28] studies how prevalence, sensitivity and specificity evolve with repeated tests albeit without a gold standard reference. They study the evolution of prevalence without compounding it between successive tests. [29] studies the evolution of sensitivity and specificity and proposes a majority rule, but does not study the compounding evolution of prevalence, which is what our work exploits. Although many articles have been published on predicting CRC using a Bayesian methodology, we could not find any papers suggesting the use of independent FITs with Bayesian deductive inference to establish the necessity of colonoscopies—the focus of this paper.

Indeed, unlike nearly all other papers on the topic, we are not trying to diagnose CRC. Instead, we propose a deductive approach to identify those who very probably do not need urgent colonoscopies, thereby enabling those at risk to access colonoscopies more quickly. Also, although many articles have been published on predicting CRC using a Bayesian methodology, we could not find any papers suggesting combining independent FITs with Bayesian deductive inference to explore its potential to reduce the number of patients on the colonoscopy waiting list—one of the key innovative ideas of our paper.

Reference [30] proposes a similar multi-faecal immunochemical testing strategy, but the authors do not suggest using Bayes’ theorem to improve inferences. While the paper describes what we want to do quite well, it uses three FITs (not four) and a frequentist analysis. This means that the authors cannot provide precise numerical estimations of the probability of CRC in their patients depending on the number of positive and negative FITs. A similar study was also performed with three FITs in [19,31], but here again, deductive or inductive Bayesian inference was not used, meaning that the conclusions of the study remain vague. Other papers also propose to perform several FITs but separated by several months, during which the state of the patient can evolve, therefore invalidating the purpose of our approach.

### 2.2. Bayesian Analysis of the Results of a FIT in the Metropolitan Region of Chile

We recall Bayes’ probability inversion theorem here because it is key to understanding the computations presented in this article. This theorem is called the “inversion theorem” because it provides the mathematically correct way to invert probabilities, which unfortunately goes against intuition.

**Establishing the “reliability” of a test:** Suppose you have invented a new pregnancy test. You can determine its specificity by conducting a clinical test on 1000 women who are known to not be pregnant. If the test returns a positive result for one of these women, then its false positive ratio is 1 in 1000, and its specificity is 99.9%.

**“Probability inversion” problem:** Knowing this 1/1000 FP ratio, what is the probability of being pregnant if the test is positive?

**Counter-intuitive answer:** Without additional information, notably, *the prior probability of the woman being pregnant,* it is impossible to tell. Indeed, the pregnancy probability could be influenced by external factors, such as whether she lives near a farm that uses organochlorine compounds as pesticides or simply her age. For example, if the woman is 20 years old, it is highly probable that she is pregnant. If she is 60 years old, her positive test result has a higher probability of being a false positive.

Bayes’ theorem provides a mathematical equation for inverting probabilities [21,32]. If testing for a sickness with a test whose reliability has previously been established, the probability of being sick if positive *P(s|p)* can be expressed as follows:

*P(s|p)* = *P(p|s)* × *P(s)/P(p)* (1)

where:

- *P(p|s)* is the *Probability* of being *Positive* if *Sick* (this is the sensitivity);
- *P(s)* is the prior *Probability* of being *Sick* (the prevalence of the sickness in the cohort to which the person belongs);
- *P(p)* is the observed *Probability* of being *Positive*.

The *P(p)* value can be confusing for non-specialists, but it is simpler than it seems. For a cohort of patients, it is the number of positive patients (i.e., the number of true positives + the number of false positives) divided by the size of the cohort.

We can see in (Equation (1)) that, in order to correctly inverse the probability, three values (called prior knowledge) are needed: *prevalence, specificity,* and *sensitivity* of the test.

#### Application to CRC Screening

A recent article [33] shows that in 2018, the age-standardised prevalence of colorectal cancer (CRC) in Chile ranges from 14.6/100,000 inhabitants in Region III (Atacama and Coquimbo) to 23.0/100,000 inhabitants in Region XII (Magallanes), with 19.6/100,000 inhabitants for Region XIII (Metropolitan area around Santiago, Chile’s capital).

If we use this prior knowledge:

- The 19.6/100,000 prevalence of CRC observed in the Metropolitan Region;
- The 73% observed sensitivity;
- The 94% specificity of a single FIT, observed on 92,447 asymptomatic people from 44 studies [9].

We can mathematically make deductions from (Equation (1)).

For every 100,000 inhabitants submitted to a single FIT:

- In probability, 19.6 are sick, but due to the 27% false negatives (FN), statistically, in probability, 5.29 of them will falsely test negative (and 14.31 will truly test positive = TP);
- In probability, 100,000 − 19.6 = 99,980.4 are healthy, but due to the 6% false positives (FP), in probability, 5998.82 of them will falsely test positive (and 93,981.58 will truly test negative = TN).

So, from the point of view of a tested person who receives a positive FIT, this person will be among 6013.13 people: the 14.31 true positives or the 5998.82 false positives. So, from (Equation (1)), a FIT positive person from Chile’s Metropolitan Region only has a 14.31/6013.13 = 0.238% chance of having CRC, which is not much.

The same principle applies to the probability of being healthy based on a negative test result. A person with a negative FIT result from Chile’s Metropolitan Region will be among 93,986.87 people: the 93,981.58 true negatives or the 5.29 false negatives, meaning they have a 93,981.58/93,986.87 = 99.995% chance of being healthy. While this is much better, it means that 56.28 out of every million FIT-negative people would be false negatives (they would think they are healthy even though they have CRC). The FIT must be improved.

### 2.3. Proposed Updated Bayesian Deduction (UBD) Method with Application to CRC

As one FIT was inconclusive due to intermittent bleeding or poor processing (making the tests independent of each other), we propose repeating the tests with a twist: because it happens that the probability of being sick within a specific population at a given time is also how prevalence is defined, we propose to divide the initial population (100,000 in our example, with a prevalence of 19.6/100,000) into two virtual subcohorts and use their computed probability of being sick as prevalence for subsequent FIT testing stages. For the example above, we can create two virtual subcohorts:

- A FIT+ virtual subcohort of 6013.132 positive people with a prevalence of 237.9/100,000;
- A FIT− virtual subcohort of 93,986.87 people with a prevalence of 5.631/100,000.

If the FIT tests are independent, we can repeat this indefinitely in a recursive manner as in the following algorithm:

1. Perform a test with known sensitivity and specificity on a cohort with known prevalence.
2. Divide into subcohorts depending on the result of the test.
3. Associate a Bayesian-computed prevalence with each subcohort.
4. Repeat from step 1.

**Figure 1:**
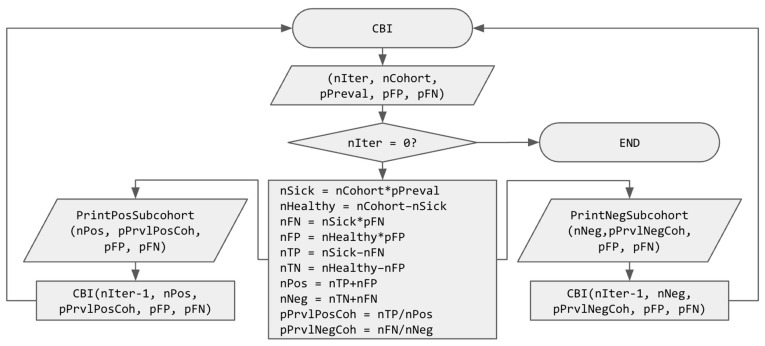
Flowchart of the recursive UBD (Updated Bayesian Deduction) function. Variable names use “*n*” as a prefix to indicate that they represent a number and “*p*” as a prefix to indicate that they represent a probability.

Figure 1 illustrates the UBD algorithm introduced in this paper with a flowchart. UBD is a recursive function, called with:

- nIter, the number of recursive calls to be computed (or, in computer science terms, the depth of the resulting tree);
- nCohort is the size of the cohort;
- pPreval is the prior known disease prevalence (in probability);
- pFP is the prior probability of a false positive result when using the test;
- pFN is the prior probability of a false negative result when using the test.

**Figure 2:**
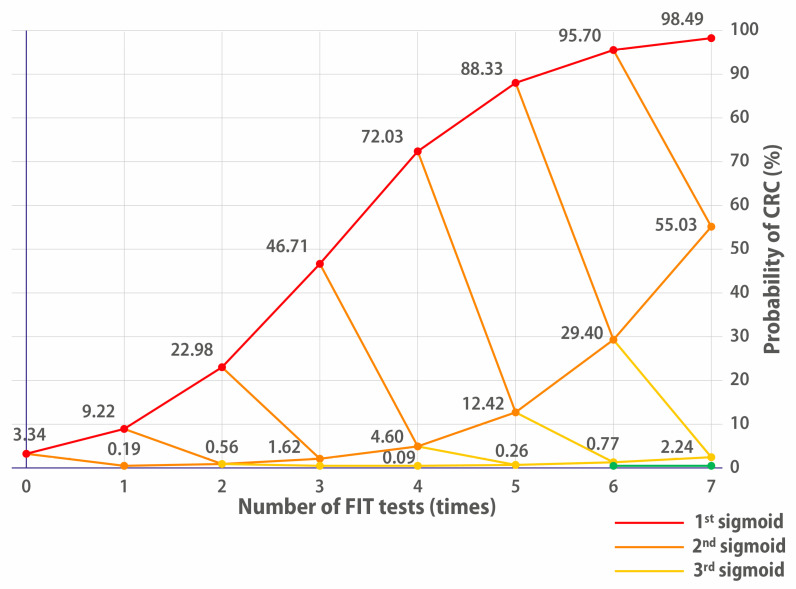
Evolution of the probability of having CRC depending on the positivity or negativity of FITs. FIT+ goes up, FIT− goes down. The red line indicates the cumulative CRC probability for people who continually have positive FIT results. Having a single negative FIT brings it to the 2nd orange sigmoid. Two negative FITs bring down the probability of CRC to the 3rd yellow sigmoid. Three negative FITs bring down the probability of having CRC to the 4th green sigmoid.

The algorithm starts with with checking if nIter = 0, in which case it ends without doing anything. Else (if the number of iterations of FIT tests is not zero), it starts computing several intermediate values, namely, the number of sick and healthy people in the cohort, the number of false negatives, false positives, true positives, and true negatives and finally, the number of positive and negative people, in order to compute the prevalence of the disease in the subcohorts of positive (pPrvlPosCoh) and negative (pPrvlNegCoh) people. The preliminary results are printed (in order to obtain the intermediary values for printing in a graph, such as Figure 2), and the UBD function is recursively called on each subcohort with one fewer iteration (nIter–1), their size (nNeg or nPos, depending on the subcohort) and updated prevalence (pPrvlNegCoh or pPrvlPosCoh). It is important to note here that pFP and pFN are not modified, but they could be updated at this point if different tests were performed or if a model of their evolution were known.

The nIter variable represents the number of FIT tests performed, i.e., the depth of the tree being developed here, since, as previously stated, the recursive procedure can be repeated nIter times because all the necessary values for each iteration are computed exactly. They are therefore available for the next recursive call of the function. Consequently, it is possible to view the results of numerous FITs on the studied cohort *without performing them in reality*, as shown in Figure 2.

The reason why repeating tests is interesting is that prevalence will compound and evolve as a sigmoid. And the interesting part of a sigmoid is not its beginning (around 0%) or its end (around 100%) but its “middle part”, where the first derivative has a high value (cf. Figure 2). The slow evolution of deduced prevalence in the beginning of the sigmoid may have caused other researchers to overlook this approach.

## 3. Results

### 3.1. Application of 4-FIT Updated Bayesian Deduction to CRC Screening in Chile’s Metropolitan Region

We propose to create two subcohorts: one subcohort with the 6013.13 FIT+ and another subcohort with the 93,986.87 FIT− people. However, because the probabilities for people of each subcohort to be sick are (thanks to Bayes’ theorem) mathematically exact, we propose to use them as the prevalence for each of the subcohorts. If we insert the initial values in the UBD algorithm of Figure 1, we can compute the following:nSick = nCohort × pPreval (100,000 × 0.000196 = 19.6)nHealthy = nCohort − nSick (100,000 − 19.6 = 99980.4)nFN = nSick × pFN (19.6 × 0.27 = 5.292)nFP = nHealthy × pFP(99,980.4 × 0.06 = 5998.824)nTP = nSick − nFN (19.6 − 5.292 = 14.308)nTN = nHealthy − nFP (99,980.4 − 5998.824 = 93,981.576)nPos = nTP + nFP (14.308 + 5998.824 = 6013.132)nNeg = nTN + nFN (93,981.576 + 5.292 = 93,986.868)pPrvlPosCoh = nTP/nPos (14.308/6013.132 = 0.00237946)pPrvlNegCoh = nFN/nNeg (5.292/93,986.868 = 0.00005631)

And we can now recursively call the UBD algorithm on the new FIT− and FIT+ subcohorts with their updated prevalence:

1. On the 6013.13 FIT+ subcohort (composed of the 5998.82 FP + 14.31 TP) with 237.9/100,000 prevalence (i.e., 11.9 higher than the original global cohort);
2. On the 93,986.87 FIT− subcohort (composed of the 93,981.58 TN + 5.29 FN) with 5.631/100,000 prevalence (i.e., 3.55 lower than before).

A prevalence of 5.631/100,000 may not seem much less than 19.6/100,000, but the effect will compound, as we will explore the evolution of the prevalence with more FITs.

We can now compute the results of Table 1.

Out of the statistical 6013.13/100,000 FIT+ patients subcohort and using the same values for specificity and sensitivity (but with an updated prevalence of 237.9/100,000) it follows that there were statistically 370.37 people who would have tested positive twice (FIT++), out of which 10.44 would have been true positives and 359.93 false positives. It also follows that there are statistically 5642.76 more positive than negative people (FIT+−), out of which 5638.89 would have been true negatives and 3.86 false negatives.

The interesting thing to see is that we now have three subgroups: 370.27 FIT++ people for whom the CRC prevalence is now 2820/100,000, 5642.76 + 5642.76 FIT+− or FIT−+ people for whom the prevalence is 68.4/100,000, and 88,344.11 FIT−− people for whom the probability of having CRC dropped from the original 19.6/100,000 down to 1.62/100,000. The probability for a FIT++ person to have CRC is still low (2.82%), but it is already increasing.

Our proposition is to repeat this more times until we reach the “middle part” of the sigmoid, where a better (number_of_FIT_tests/significative_probability) ratio can be found.

Table 2 shows the theoretical results of performing four consecutive FITs on 100,000 inhabitants of the Metropolitan Region. Because the prevalence of the previous subcohort is used to compute the prevalence of the next subcohort and because the prevalences compound, we keep a large number of decimals for representing the prevalence (while we are rounding down to two decimals for the number of concerned patients). Indeed, these numbers are intermediary values in between two deductions.

Looking at Table 2, we see that 6.86 + 89.43 = 96.29 of the 100,000 people urgently need a colonoscopy: the 6.86 FIT++++ because they have a >81% probability of having CRC. Then, 89.43 FIT+++− people have a still life-threatening 9.2% chance of having CRC (469 times higher than the original prevalence). The 19.6 people who statistically have CRC in this cohort of 100,000 could be among them.

Performing 96 colonoscopies (rather than 100,000, as would be performed in Germany) reduces the number of colonoscopies by 99.9%. While this makes high-quality CRC screening more affordable for many countries, it also necessitates the performance of four FITs on 100,000 people. Even though FITs are much cheaper, non-invasive, and readily available and do not require any significant infrastructure, the difficulty of organising a population screening based on four FITs must not be underestimated. However, this should be compared with the difficulty of organising a full population colonoscopy screening.

Are the 19.6 people in the 96 people with a >9% probability of having cancer?

There are 1913 FIT++−− people who have a 0.24% chance of having CRC, which is 12.25x more than the original observed prevalence of 0.0196%. But here again, 1913 additional colonoscopies could be performed much more easily than 100,000. An alternative could be to offer these at-risk people more FITs (still cheaper and easier to do than a colonoscopy) to discern who needs a colonoscopy and who does not to find all 19.6 people who statistically have CRC.

There are 19,931.24 FIT+−−− people who have a 0.005652% chance of having CRC, which is 3.47 times less than the original asymptomatic population of the Metropolitan Region (which is currently not screened in Chile). They could be tested again in following years.

Finally, 78,059.7 FIT−−−− people have virtually 0% chance of having CRC. It may not be necessary to test them in the near future. We show in Figure 2 of Section 3.2.1 that, even on a symptomatic cohort, many more FITs would be needed to bring other significant results, so this is why we think that four tests is an optimum value for faecal immunochemical tests.

As a conclusion, a UBD CRC screening with four FIT without-waiting-list tests:

- Singles out only 96 high-risk people out of 100,000 to whom a colonoscopy could be prescribed;
- Shows that 78,058 colonoscopies could be avoided and possibly 19,931 more, resulting in the possibility of replacing 98.99% (or even 99.9%) of colonoscopies with 4xFITs.

Cost Evaluation of Implementing a 4-FIT UBD Screening on 100,000 People

If we take a cost of USD 5 for a FIT and USD 500 for a colonoscopy [3], screening the whole 100,000 cohort would cost USD 50,000,000 and would be very difficult, if not impossible, to perform rapidly without adequate infrastructure and personnel.

Performing a 4-FIT UBD screening protocol and the necessary 96 urgent colonoscopies would cost 100,000 × 4 × 5 + 96 × 500 = only USD 2,048,000 compared to USD 50,000,000 for a full population colonoscopy screening with no associated waiting list. A total of 96 high-risk patients would be identified and their treatment would be started immediately. If judged necessary, performing a colonoscopy on the other 1913 FIT++−− people could be organised and would cost an additional USD 956,500 They could easily be performed within months in the public hospitals of the Metropolitan Region.

### 3.2. Result of the Application of Four Consecutive FIT Updated Bayesian Deduction (4-FIT UBD) on a Symptomatic Cohort

A recent study on 1315 Chilean symptomatic patients [34] observed the predictive quality of a single FIT for colorectal cancer with colonoscopy validation. A total of 507 patients were removed from the FIT study: those who had previously been diagnosed with CRC and 345 patients who were prioritised for a colonoscopy due to their symptom-based classification as high-risk (HR) patients (cf. Section 3.2.2). The remaining patients (808 Low-to-Moderate Risk patients) were prescribed a FIT, which they returned before the validation colonoscopy. Table 3 (a replicate of Table 2 of the study) gives us all the necessary data to infer the results that can be obtained by a 4-FIT Updated Bayesian Deduction method. The three pieces of prior information needed for Bayesian Inference (*prevalence, test specificity,* and *sensitivity*) can be computed from Table 3:

- A total of 27 were diagnosed (with colonoscopy) with CRC, yielding a prevalence of 3.3415841584% (27 out of 808), i.e., 170 times higher than for the asymptomatic cohort. Please note that we get exact numbers because we exploit exact observed values by deductive inference, and not inductive inference. We keep a large number of decimals because the probabilities will compound, so rounding to two decimals will be a source of future errors. This also guarantees the reproducibility of the presented results.
- Out of 781 non-CRC patients, 256 were FP = 67.2215108835% specificity.
- Out of the 27 CRC patients, 1 was FN = 96.2962962963% sensitivity.

FP and FN values are very different from those published in the literature for FITs because, generally, studies are performed on asymptomatic patients for screening purposes. However, as stated in [34], CRC symptoms are nonspecific: rectal bleeding can come from haemorrhoids or ulcerative colitis or other factors that may not be cancerous tumours. So, if occult blood in stool is what is tracked for CRC detection, more false positives will be found in “symptomatic patients” than in an asymptomatic population. We now see that the specificity of the test does not only depend on the test itself, but it also depends on the cohort. However, sensitivity will not be affected. This is important if the objective is to detect who does not have CRC.

Using these values, we can infer the results of more FITs on this specific cohort (cf. Table 4).

We see that the probability of being sick if testing positive (FIT+) is 9.22%, which is 2.76 times higher than in the original cohort. The probability of being sick if tested negative (FIT−), is now 0.19%, so the subcohort prevalence has been divided by 17.58. Unfortunately, a 0.19% chance of having CRC is still much too important, because it is still 10 times higher than the 0.0196% of the Metropolitan Region to which they belong: for every 1000 patients, 2 patients will unknowingly have CRC.

As for the simple example, our proposition is to now consider two subcohorts: one cohort of 308 FIT+ patients and a second cohort of 500 FIT− patients, noting that every time a FIT is performed on a subcohort, the prevalence rates are updated depending on the positivity or negativity of the test, with the probability of having CRC found in the previous cohort.

#### 3.2.1. Results of 4 FITs on the Low/Moderate Risk Cohort of [34]

We now present both an aggregated table for the 4-FIT Updated Bayesian Deduction in Table 5 and Figure 2, which represents the sigmoid evolution of the probability for patients to have CRC over multiple consecutive tests:

The 32 FIT++++ patients must clearly be prioritised for a colonoscopy, as they have a 72% chance of having CRC; they are on the first sigmoid of Figure 2. The 4xFIT UBD screening shows its efficiency here because they would be immediately prioritised (this is already what the team of [34] has achieved on their high-risk priority patients, who benefited immediately from a colonoscopy).

The 77 FIT+++− patients who had a 4.6% chance of having CRC could also have been prescribed a colonoscopy because they were on the second orange sigmoid of Figure 2. Altogether, the cumulated number of FIT++++ and FIT+++− patients of the Low/Moderate Risk cohort was only 109/808.

One could think that the 228 FIT++−− patients were more problematic because they had a 0.09% chance of having CRC, which is 4.6 times more than the prevalence of CRC in the asymptomatic population of the Metropolitan Region to which they belong.

However, Figure 2 shows us that even if they had been positive for a fifth FIT, this would not have been conclusive. The third (pale yellow) sigmoid evolves really slowly. The decision of whether or not to lower their priority in accessing colonoscopy could lie with the hospital ethics committee. Figure 2 shows that performing a fifth or sixth test would not have yielded much additional significant information, showing a limit of the multiple FITs for this cohort. The probability % increases somewhat, but the gain is not worth the extra expense. This is why we posit that, for this cohort, four FITs would have represented the optimal balance between significance and number of tests.

The 470 FIT+−−− and FIT−−−− patients had a really low probability of having CRC (less than 0.002%, i.e., 10 times less than the prevalence of CRC in the asymptomatic people of the Metropolitan Region to which they belong). Their access to a colonoscopy could probably have been safely lowered in spite of their initial belonging to the Low to Moderate Risk cohort, therefore giving the opportunity to other higher-risk patients to have had access to a colonoscopy sooner.

To summarise, we posit that four FITs would indicate whether patients from an identical cohort would be on the first two “dangerous sigmoids”. If patients on the first and second sigmoid (FIT++++ and FIT+++−) had been prioritised, their number would be 109/808, which means that their waiting list could have been reduced by 86.5%.

#### 3.2.2. Potential Results of Four FIT UBD on the High-Risk Patients Cohort [34]

Reference [34] also gives us the prevalence of CRC in the high-risk cohort: it was composed of 345 patients, of which 16 were diagnosed with CRC thanks to a high-priority colonoscopy. Because they were patients of the same original cohort, we could assume that they would have been tested the same way as the L/MR cohort. So, even though the specificity of the FIT would be lower (due to the fact that they were more symptomatic), the important thing in this study is the sensitivity of the test, which has no known reason to vary.

Table 6 shows the aggregated results for the high-risk cohort.

Had a 4-FIT UBD protocol been used on this cohort, 51 patients could have been prioritised. A total of 294 out of 345 patients could have been given a lower colonoscopy priority, reducing the waiting list for FIT++++ and FIT+++− patients by 85.2%.

More importantly, these 294 patients had a CRC probability of ≤0.127% compared to 109 patients in the L/MR cohort who had a probability of CRC of >4.6%. Their five-year survival rate could have been improved had they not had to wait for the 294 ≤ 0.127% patients to have their colonoscopies before them. Ten of the L/MR patients died between the study date in 2019 and the paper submission date in March 2025.

### 3.3. Mathematical Analysis of the Performance of Multiple FIT Updated Bayesian Deduction Tests on the L/MR Cohort of [34]

**Figure 3:**
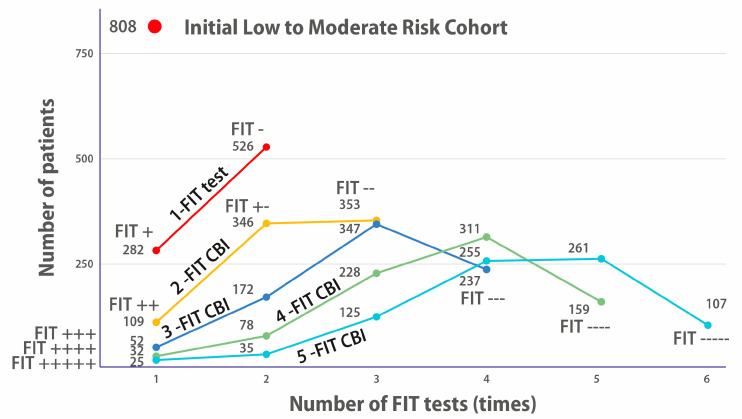
Evolution of the number of positive or negative patients for 1 to 5 FIT UBD tests on the L/MR cohort. The red dot on top shows the size of the original cohort of 808 Low–Moderate symptomatic patients. The orange line shows the number of patients who, on a single FIT, were positive (282 FIT+) or negative (526 FIT−). The yellow line shows how many would have been FIT++, FIT+−, or FIT++ had they been proposed to do 2 FITs. The dark blue line shows how many would have been FIT+++, FIT++−, FIT+−−, and FIT−−− had they been proposed to do 3 FITs and so on for 4 and 5 FITs.

Figure 3 is very interesting because it shows the evolution of the number of positive or negative patients for one to five FIT UBD tests on [34]’s L/MR cohort.

After one FIT is performed (top dark orange curve), two categories appear: 282 positives and 526 negatives. However, we know that only 27 patients of the 808 have been diagnosed for CRC. So, the 282 positives reflect the imprecision of a single test (too low specificity).

As the number of tests increases (2-FIT UBD, 3-FIT UBD, *etc*.), we see that there are fewer and fewer continuously positive patients: 109 FIT++ (in a 2-FIT), 52 FIT+++ (in a 3-FIT) *etc*., until, for five FITs, we see that there are only 25 FIT+++++, even though we know that there were 27 diagnosed patients.

We see here the imprecision linked to the imperfect sensitivity of looking for occult blood in faeces for detecting CRC patients: *over five FITs, 2 of the 27 diagnosed patients had at least one negative FIT.* This directly corroborates that the bleeding of polyps is intermittent.

*The hope that increasing the number of FITs will precisely detect the number of sick patients is illusory due to intermittent bleeding.*

There will always be patients with CRC who will have negative FITs: the more tests performed, the greater the likelihood that some tests will be false negatives.

Plotting the number of people with positive and negative FITs shows, in a different way, why four consecutive tests seem to be the optimal number for FITs. If we wish to only perform colonoscopies on FIT-positive patients:

- With a 3-FIT, there were 52 FIT+++ patients. A total of 52 colonoscopies would be performed to find the 27 who had CRC.
- A 4-FIT reduces the colonoscopies to 32 FIT++++patients.
- But with five FITs, there are only 25 FIT+++++, when we know for a fact that there are 27 CRC patients, so we are missing 2.

If colonoscopies had been performed on both the 25 FIT+++++ and 35 FIT++++− patients of a 5-FIT UBD protocol, 25 + 35 = 60 colonoscopies would have been performed, which is more than the number of colonoscopies for the 3-FIT 52 FIT+++ patients and more than for the 4-FIT 32 FIT++++ patients.

Due to intermittent bleeding, performing five FITs would (on this cohort) lead to more colonoscopies than with four tests only.

## 4. Discussion

It is interesting to note that “*The current organized screening program for colorectal cancer in Germany offers both sexes 5 annual fecal immunochemical tests (FITs) between ages 50 and 54 years, followed by a first screening colonoscopy at age 55 years if all of these FITs were negative*” [35]. One of the findings of the paper is the following: “*Although annual FITs at ages 50–54 may contribute to earlier diagnosis of CRC, the current sequence of CRC screening, if fully adhered to, may not use screening capacities in the most efficient manner*”.

The problem with “*consecutive FITs at ages 50–54 as currently offered in the German cancer early detection program*” is that, even though this means five FITs, they cannot be “combined” to increase (or decrease) probabilities, as presented in this paper. Indeed, during the time between two FITs, cancer can evolve. If it was not detected by the first FIT (possibly because early-stage polyps do not bleed constantly), then, if it is diagnosed the next year, it could be found that the FIT of the first year did not help detect the cancer that developed.

The 4-FIT UBD approach we present in this paper is challenging for CRC screening, as collecting four separate samples could be difficult to implement, both for the patients and the hospitals. However, in the current full-colonoscopy alternative, *Germany also performs five FITs (albeit separated by one year)*. The number of countries that can afford five FITs plus a colonoscopy is very low, as is adherence to colonoscopy: apparently, in Germany, only 23% of males and 24% of females perform their prescribed colonoscopy over 10 years [36].

Some participants even choose not to undergo a colonoscopy following a positive test result for various reasons, among which are “*Practical barriers, Discomfort of the examination, Personal integrity, Multimorbidity, Feeling healthy, Not having the energy*” and literally 36 other bad reasons for avoiding a colonoscopy [37]. Therefore, despite the fact that patient compliance and logistics are important factors that make 4-FIT UBD an imperfect solution for CRC screening, Germany proposes five inconclusive FITs plus a colonoscopy.

For dealing with symptomatic patients on a hospital waiting list for a colonoscopy, the 4-FIT UBD protocol could be proposed to patients and medical teams as a cheap and innocuous way to better prioritise (or even avoid) colonoscopies. Once, for a particular hospital, the three prior knowledge values (*prevalence*, *specificity*, and *sensitivity* of the FIT used in the hospital) have been determined with confidence, patients on the colonoscopy waiting list could be efficiently prioritised knowing that other criteria (such as age, sex, presence of anaemia, *etc.*) can be added in the Updated Bayesian Deduction graph to potentially better adapt to a specific patient.

### Combining Other Indicators (Such as Age) with Multiple FITs

Supposing that we have a cohort of 1000 patients, of whom 100 have CRC, the CRC prevalence in the whole cohort is, therefore, 10%. Then, suppose we observe that, out of 400 patients between 65 and 75, 60 of them have CRC, the prevalence in the [65, 75] year-old patients is 15%. But we can also deduce that the [65, 75] age indicator has a false positive percentage of 85%, because if “*being between 65 and 75 is a CRC indicator*”, then 340 were positive to this indicator but did not have CRC, so the false positive percentage is 340/400 = 85%. The false negative ratio can be easily computed as well: 600 were “negative” because they were younger than 65 or older than 75; but nevertheless, 40 of them had CRC. So, the false negative ratio is 40/600, i.e., 6.67%. These three values (prevalence of 15%, 85% FP, and 6.67% FN) are undisputable because all are mathematically deduced from the observed data. In this sense, the Bayesian deduction is purely “evidence-based”.

What can be discussed is whether deductive inference has any predictive value. If a new patient aged 70 comes in, does it make sense to tell them that by looking at the statistics, they have a “15% chance of having CRC”? Many statistical studies associate a “confidence interval” to such inferred information, but it is important to look at what Wikipedia says about confidence intervals: “*A 95% confidence level does not imply a 95% probability that the true parameter lies within a particular calculated interval. The confidence level instead reflects the long-run reliability of the method used to generate the interval*” (https://en.wikipedia.org/wiki/Confidence_interval (accessed on 13 October 2025)).

This means that the confidence interval is of the same nature as Bayesian Inference: it reflects the past. *It is descriptive and not predictive*. According to the numbers above, patients outside of the [65, 75] age interval have a 40/600, i.e., 6.67% chance of having CRC. However, we know that the probability of a 20-year-old patient having CRC is much lower than 6.67%. Humans are too complex to be reduced to a handful of numerical values. It is here that the experience of a medical team and an ethics committee must play a role.

The results of 4-FIT Updated Bayesian Deduction should be understood as what they are, i.e., mathematically indisputable *deductive* inferences. We did not provide confidence intervals for this study because they are as deductive in nature as is the pure Bayesian Inference.

## 5. Conclusions and Future Perspectives

This research aims to demonstrate that thanks to the Bayesian Compound Inference, four inexpensive (and immediate) FITs could provide valuable additional help for medical teams to prioritise CRC symptomatic patients for a colonoscopy. We show in the last paragraph of Section 3.2.2 that even though the symptoms-based assignment of patients to a high-risk cohort or a Low to Medium Risk cohort by the team of [34] was very efficient, prioritising all patients with a 4-FIT BCI approach could have been more optimal.

Using this approach could have a huge impact in countries such as Chile where, in public hospitals, symptomatic patients could wait more than a year for a colonoscopy.

If used for screening, 4-FIT UBD could be transformative because we show that if it were implemented in Chile’s Metropolitan Region, only 96 out of 100,000 people in Chile’s Metropolitan Region would be prescribed an urgent colonoscopy in order to detect the 19.6 people who statistically have CRC [33]. Finding these people early could drastically improve their five-year survival chances and drastically reduce the number of urgent colonoscopies by screening for CRC with 4x FITs rather than colonoscopies, which would make high-quality general population screening much more affordable for low- and medium-revenue countries.

Finally, the Updated Bayesian Deduction can:

- Be multimodal by integrating other indicators such as sex or age (as shown in the discussion) or blood test markers [38] to better match the patient;
- Also be used for screening (or prioritising resources for) other types of cancer or diseases. The number of mammograms could possibly be reduced if cheaper, less invasive, and less resource-intensive independent tests, such as blood markers for breast cancer, are available [39];
- Not only save lives but also money, as the reason for resource scarcity is typically their overall cost in equipment and human resources.

Improving the quality of information with Updated Bayesian Deduction could allow doctors to use resources more effectively and save lives, as well as drastically reducing the cost of colorectal cancer care, even in wealthy countries.

However, as discussed in the Discussion section of this paper, deductive statistics (and even inductive statistics with confidence intervals) should not be the exclusive source of decisions. A more holistic approach to the patient should be taken, involving doctors and a medical team, with the help of an ethics committee.

We hope that applying Updated Bayesian Deduction to repeated independent tests could contribute to improving not only CRC detection and treatment but also general health care around the world.

## Figures and Tables

**Table 1:** Theoretical result of 2 consecutive FITs on Metropolitan Region patients. (Colour code evolves from green for 0% CRC probability to dark pink for 4% probability to black for over 50% CRC probability).

**Second FIT test**				
				
**ON FIT+ people**	Cohort size	Specificity %	Sensitivity %	Prevalence
	6013.13	6.00	27.00	0.237946%
	Concerned	True	False	Sickness prob.
**FIT ++**	370.37	10.44	359.93	**2.820077%**
**FIT +–**	5642.76	5638.89	3.86	**0.068462%**
				
**ON FIT–people**	Cohort size	Specificity %	Sensitivity %	Prevalence
	93,986.87	6.00	27.00	0.005631%
	Concerned	True	False	Sickness prob.
**FIT -+**	5642.76	3.86	5638.89	**0.068462%**
**FIT --**	88,344.11	88,342.68	1.43	**0.001617%**

**Table 2:** Aggregated result of 4 consecutive FITs on Chile’s Metropolitan Region population. (Colour code evolves from green for 0% CRC probability to dark pink for 4% probability to black for over 50% CRC probability).

**4-FIT Test**				
	**FR %**	**FN %**	**Cohort Size**	**Sick**
**On 3-FIT** **subcohorts**	6.00	27.00	100,000.00	**19.60**
	Concerned	True	False	Sickness prob.
**FIT++++**	6.86	**5.57**	**1.30**	**81.116533%**
**FIT+++−**	89.43	**8.23**	**81.20**	**9.207504%**
**FIT++−−**	1912.77	**4.57**	**1908.20**	**0.238846%**
**FIT+−−−**	19,931.24	**1.13**	**19,930.11**	**0.005652%**
**FIT−−−−**	78,059.70	**0.10**	**78,059.59**	**0.000133439%**

**Table 3:** Diagnostic accuracy of qualitative faecal immunochemical test (replicate of Table 2 of [34] of the results of one FIT on the Low/Moderate Risk cohort).

Variable	Total (*n* = 808)
FIT outcomes; *n* (%)	
*True positives*	26
*True negatives*	525
*False positives*	256
*False negatives*	1
*CRC diagnosis confirmed*	27
Qualitative FIT; % (confidence interval 95%)	
*Sensitivity*	96.3 (81.71–99.34)
*Specificity*	67.22 (63.85–70.42)
Predictive value % (confidence interval 95%)	
*Positive (PPV)*	9.22 (6.37–13.17)
*Negative (NPV)*	99.81 (98.93–99.97)
Likelihood ratio	
*Positive (LR+)*	2.93
*Negative (LR−)*	0.06

**Table 4:** Finding out the new prevalence of CRC in the FIT+ and FIT– subcohorts. (Colour code evolves from green for 0% CRC probability to dark pink for 4% probability to black for over 50% CRC probability).

**Parameters**	**Cohort size**	**Prevalence %**	**Specificity %**	**Sensitivity %**
	808	3.34	67.22	96.30
				
**First FIT test**				
	Specificity %	Sensitivity %	Cohort size	Sick
**Original cohort**	32.78	3.70	808	27.00
	Concerned	True	False	Sickness prob.
**FIT +**	282.00	26.00	256.00	**9.219858%**
**FIT -**	526.00	525.00	1.00	**0.190114%**

**Table 5:** Cumulated results of four FITs on the L/MR cohort. (Colour code evolves from green for 0% CRC probability to dark pink for 4% probability to black for over 50% CRC probability).

4-FIT Test				
	FR%	FN%	Cohort Size	Sick
**On 3-FIT** **subcohorts**	32.78	3.70	808.00	**27.00**
	Concerned	True	False	Sickness prob.
**FIT++++**	32.23	**23.22**	**9.02**	**72.028812%**
**FIT+++−**	77.53	**3.57**	**73.96**	**4.607010%**
**FIT++−−**	227.71	**0.21**	**227.51**	**0.090493%**
**FIT+−−−**	311.05	**0.01**	**311.05**	**0.001699%**
**FIT−−−−**	159.47	**0.00**	**159.47**	**0.000031858%**

**Table 6:** Cumulated results of four FITs on the HR cohort. (Colour code evolves from green for 0% CRC probability to dark pink for 4% probability to black for over 50% CRC probability).

4-FIT Test				
	FR%	FN%	Cohort Size	Sick
**On 3-FIT** **subcohorts**	32.78	3.70	345.00	**16.00**
	Concerned	True	False	Sickness prob.
**FIT++++**	17.56	**13.76**	**3.80**	**78.366618%**
**FIT+++−**	33.27	**2.12**	**31.16**	**6.361622%**
**FIT++−−**	95.96	**0.12**	**95.84**	**0.127253%**
**FIT+−−−**	131.03	**0.00**	**131.03**	**0.002390%**
**FIT−−−−**	67.18	**0.00**	**67.18**	**0.000044816%**

## Data Availability

Data can be found in the Supplementary Materials.

## Supplementary Materials

The following supporting information can be downloaded at: https://www.mdpi.com/article/10.3390/cancers17233845/s1, Table S1: Speadsheet implementation for the result of 5 consecutive FIT tests for CRC detection.
